# Googling Musculoskeletal-Related Pain and Ranking of Medical Associations’ Patient Information Pages: Google Ads Keyword Planner Analysis

**DOI:** 10.2196/18684

**Published:** 2020-08-14

**Authors:** Yoichiro Yamaguchi, Deokcheol Lee, Takuya Nagai, Taro Funamoto, Takuya Tajima, Etsuo Chosa

**Affiliations:** 1 Department of Orthopaedic Surgery Faculty of Medicine University of Miyazaki Miyazaki Japan

**Keywords:** Google, ad words, infodemiology, musculoskeletal-related pain, patient education, medical information

## Abstract

**Background:**

Most people currently use the internet to obtain information about many subjects, including health information. Thus, medical associations need to provide accurate medical information websites. Although medical associations have their own patient education pages, it is not clear if these websites actually show up in search results.

**Objective:**

The aim of this study was to evaluate how well medical associations function as online information providers by searching for information about musculoskeletal-related pain online and determining the ranking of the websites of medical associations.

**Methods:**

We conducted a Google search for frequently searched keywords. Keywords were extracted using Google Ads Keyword Planner associated with “pain” relevant to the musculoskeletal system from June 2016 to December 2019. The top 20 search queries were extracted and searched using the Google search engine in Japan and the United States.

**Results:**

The number of suggested queries for “pain” provided by Google Ads Keyword Planner was 930 in the United States and 2400 in Japan. Among the top 20 musculoskeletal-related pain queries chosen, the probability that the medical associations’ websites would appear in the top 10 results was 30% in the United States and 45% in Japan. In five queries each, the associations’ websites did not appear among the top 100 results. No significant difference was found in the rank of the associations’ website search results (*P*=.28).

**Conclusions:**

To provide accurate medical information to patients, it is essential to undertake effective measures for search engine optimization. For orthopedic associations, it is necessary that their websites should appear among the top search results.

## Introduction

### Justification

Searching for information on the internet is now common practice. Medical information is no exception, and the term “consulting Dr. Google” has been popularized to represent searching for medical information on the internet [1]. Various studies have investigated the reliability and accuracy of medical information acquired on the internet [2-4]. However, there has been no research to specifically examine the extent to which medical information from websites supervised by medical associations is acquired from search results.

### Background

As of 2018, more than 100 million people in Japan (79.8% of the total population) used the internet with various devices, including half the population aged 65-79 years [5]. Japan is an increasingly aging society, and many older people suffer from locomotive disorders [6]; thus, it can be assumed that these individuals use the internet to obtain information about their conditions. According to one survey, 21.2% of patients referred to medical information from a medical institution on the internet, and 12.1% of patients referred to the social networking services of a medical institution or the government before going to the hospital [7].

Among all currently available search engines, Google has an overwhelming market share in Japan, accounting for 91.2% of searches on computers and 99.1% of searches on mobile devices [8]. Many people with musculoskeletal pain such as lower back pain and sciatica use Google to obtain information about their conditions. In such situations, it is valuable to know the type of information that people actually obtain from internet searches. Many hospitals, companies, public interest groups, bloggers, and celebrities have posted medical information on websites. Accordingly, a substantial amount of inaccurate information about medical health has been posted globally [9]. We work in the orthopedic surgery department of a hospital and encounter many patients who are misled by inaccurate medical information about musculoskeletal-related pain. The Japan Medical Association states that it will support the health and cultural well-being of the Japanese population. Providing accurate medical information to patients is one of the missions of such medical associations, and orthopedic associations play a key role in the provision of online information about musculoskeletal-related pain.

Orthopedic associations have their own websites with patient education resources that provide information on symptoms and diseases. However, it is not known whether these websites provided by the orthopedic associations appear in the results of searches with search engines. Google Trends and Google Ads are often used to investigate what searches are actually performed on the internet. A relationship between Google’s search volume increase and the influenza epidemic was noted based on such analyses [10-12]. In this study, we used the same tools to verify actual search queries and their relationship to orthopedic associations’ website ranks. We also investigated seasonally varying varieties of musculoskeletal-related pain queries that were frequently searched. In addition, we investigated whether there is a more appropriate season for providing effective medical information for each symptom related to musculoskeletal-related pain. Based on this information, we investigated whether the associations’ websites were able to provide suitable medical information to patients.

## Methods

### Search Strategy for Data Extraction

We extracted research volumes for the term “pain” (Japanese: “itami”) and generated keywords using Google Ads Keywords Planner. The data were generated separately for each region: the United States (in English) and Japan (in Japanese). The data were collected in June 2020 and Google was used for all searches. The planner generated a list of proposed terms associated with “pain” and expressed the search volume for every month. The monthly search volumes for all keywords were extracted from June 2016 to December 2019. Total search volumes for the 4 years were calculated by population ratio and sorted in descending order. Among the generated keywords, two orthopedic doctors chose musculoskeletal pain–related keywords independently. The final list was refined by the senior author (EC). From this list, the top 20 search keywords were selected. Google searches were performed with the top 20 keywords and total hit websites were recorded. We employed Google Chrome without logging in to a Google account; we blocked location information and did not use the autocomplete function. We conducted the search in incognito mode. When searching, a proxy server was used. The search was performed through servers in Japan and the United States for each language. The Japanese Orthopedic Association (JOA) patient information pages [13] and the American Academy of Orthopedic Surgeons (AAOS) patient education pages [14] were selected as the association websites. The results of the searches were checked for the top 100 sites and those not displayed within the top 100 were ranked as over 100.

### Statistical Analysis

All pain-related suggested searches in a 4-year period in both Japan and the United States were collected, and the number of searches per 100 people in each country was calculated. The monthly average number of musculoskeletal-related pain searches from June 2016 to December 2019 was also calculated. Among the keywords ranked within the top 20, those common to the United States and Japan were compared with seasonal variations. The Kruskal-Wallis test, Mann-Whitney *U* test, and Spearman rank correlation coefficient were used for statistical analysis of the search results. *P* values of <.05 were considered to indicate a significant difference. Statistical analyses were performed using Statistical Package for the Social Sciences (IBM Corp Released 2012, IBM SPSS Statistics for Windows, Version 21.0. Armonk, NY, USA).

## Results

**Figure 1:**
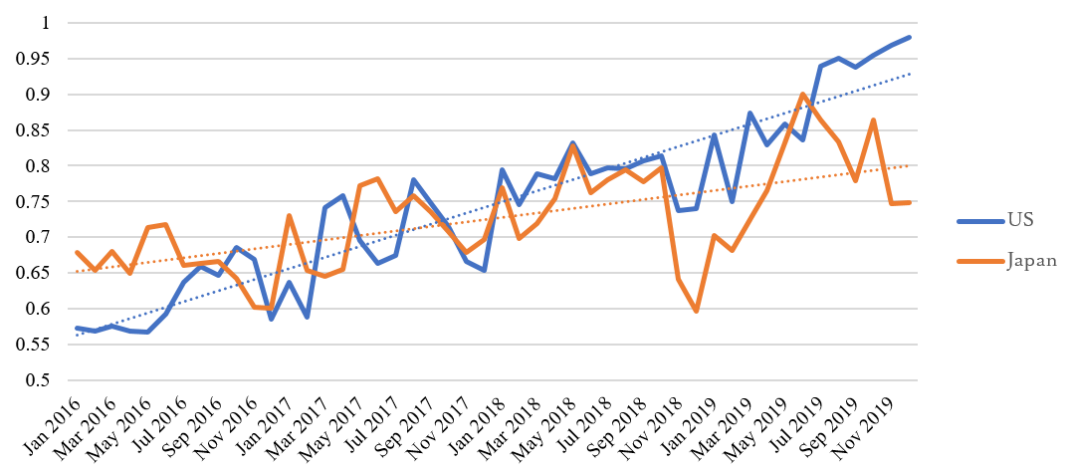
Total number of musculoskeletal-related searches in the United States and Japan (total monthly search volume of the top 20 search queries). Vertical axis: Searches per 100 people.

**Figure 2:**
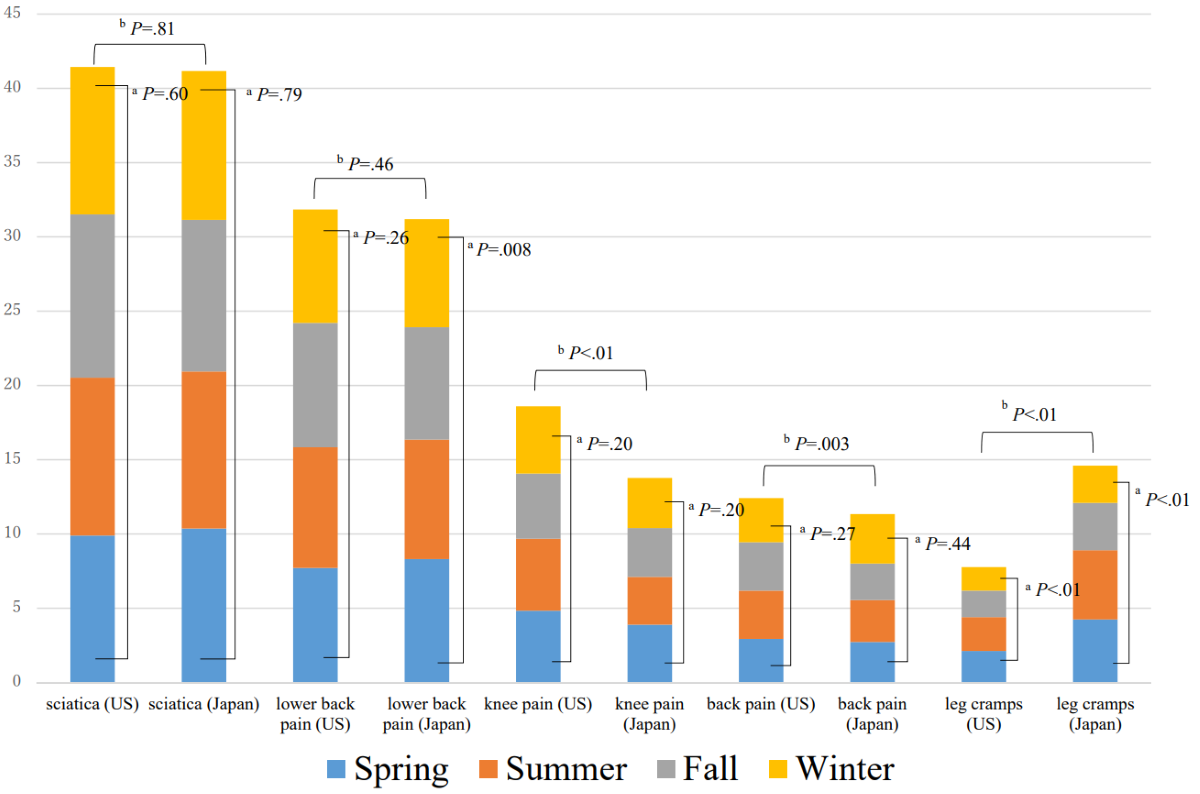
Comparison of musculoskeletal pain–related searches per month for each season (2016-2019) Vertical axis: Monthly searches per 10,000 people. ^a^Differences between seasons (Kruskal–Wallis test); ^b^Differences between the United States and Japan (Mann–Whitney U test).

**Figure 3:**
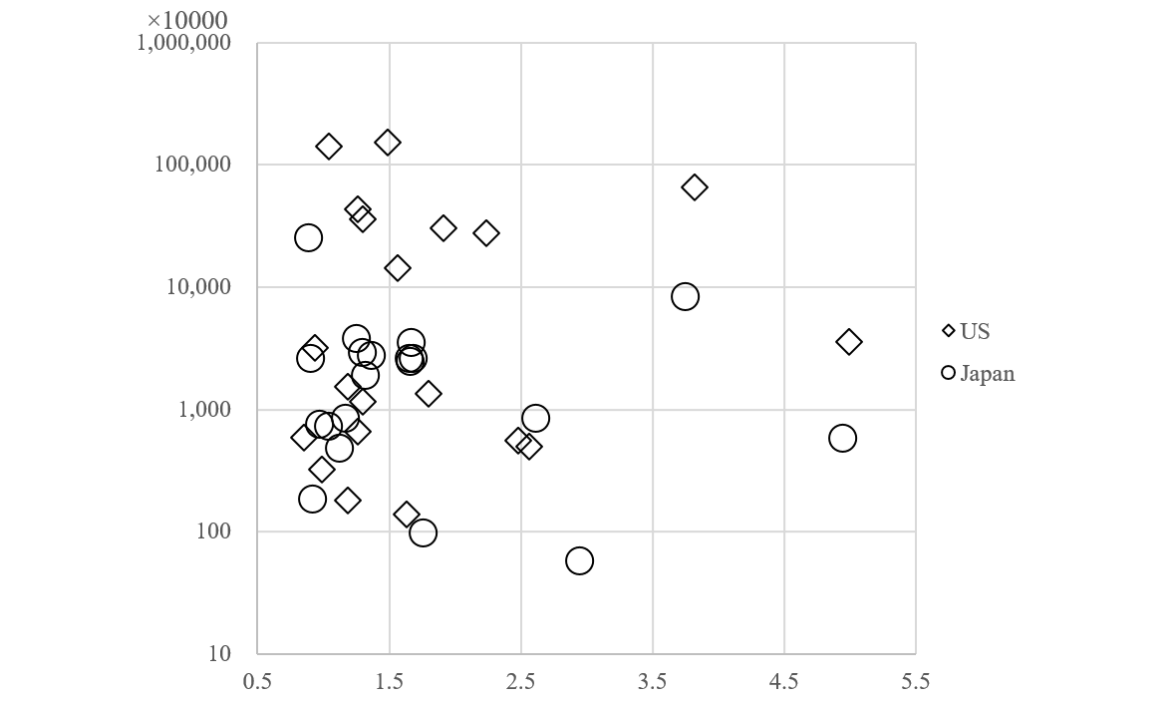
Search volumes and number of total hit websites. Vertical axis: Number of total hit websites (logarithmic scale). Horizontal axis: Four-year search volume per 100 people. No significant difference was observed in the relationship between the search volume and the number of total hit websites (Spearman rank correlation coefficient=0.13).

The total number of generated keywords from “pain” was 930 in the United States and was 2400 in Japan. In Japan, the search term was “itami,” a word that contains a Chinese character and Hiragana. In all generated queries, the average search volume ranged from 90 to 301,000 per month in the United States and from 140 to 135,000 per month in Japan. The search volume of the top 20 musculoskeletal pain–related queries ranged from 60,500 to 301,000 per month in the United States and from 22,200 to 135,000 per month in Japan. The population ratio was 0.85-4.99 per month per 100 people in the United States and was 0.88-4.94 per month per 100 people in Japan. No significant difference was found between the number of searches per population for the two countries (Mann-Whitney *U* test *P*=.67). Regarding the association websites that appeared within the top 100, the average rank was 16.47 (1st to 35th, mean 17th) in the United States and was 24.47 (2nd to 95th, mean 7th) in Japan. Within the top 20 queries, the association websites ranked within the top 10 for 30% (6/20) of queries in the United States and for 45% (9/20) of queries in Japan. No significant difference was found between the number of ranked websites within the top 10 (Mann-Whitney *U* test *P*=.28). The total search volume of the top 20 queries was 0.75 per 100 people in the United States and was 0.73 per 100 people in Japan (Table 1 and Table 2). As shown in Figure 1, the total search volume increased year after year. In the top 20 queries, the most common search queries in the United States and Japan were “sciatica,” “lower back pain,” “back pain,” “knee pain,” and “leg cramps” (Figure 2). Seasonal variation in the number of searches was found for “leg cramps,” which was significantly higher in the summer in both the United States and Japan. No significant difference was found between search volume and the number of total hit websites for the top 20 queries (Figure 3).

**Table 1:** Top 20 search queries in the United States.

Search queries	Average search volume per month	Four-year total search volume (per 100 people)	Total hit websites	Association website rank
sciatica	301,000	15,491,000 (4.99)	35,400,000	35
lower back pain	246,000	11,862,000 (3.82)	664,000,000	28
herniated disc	165,000	7,957,500 (2.56)	5,010,000	7
bursitis	165,000	7,687,000 (2.48)	5,610,000	8
knee pain	135,000	6,926,000 (2.23)	278,000,000	12
shoulder pain	135,000	5,941,000 (1.91)	302,000,000	7
carpal tunnel syndrome	110,000	5,594,000 (1.80)	13,400,000	1
muscle relaxers	110,000	5,043,500 (1.62)	1,370,000	>100
heel pain	110,000	4,869,000 (1.57)	143,000,000	6
back pain	90,500	4,624,000 (1.49)	1,510,000,000	27
neck pain	90,500	4,044,000 (1.30)	364,000,000	19
muscle spasm	90,500	4,038,000 (1.30)	11,400,000	25
hip pain	74,000	3,918,000 (1.26)	432,000,000	17
sciatic nerve pain	74,000	3,910,400 (1.26)	6,680,000	>100
piriformis syndrome	74,000	3,680,000 (1.19)	1,800,000	>100
heel spur	74,000	3,676,500 (1.18)	15,500,000	7
foot pain	60,500	3,237,000 (1.04)	1,440,000,000	>100
myalgia	60,500	3,062,500 (0.99)	3,240,000	>100
leg cramps	60,500	2,892,500 (0.93)	31,700,000	23
hip bursitis	60,500	2,639,600 (0.85)	5,910,000	25

**Table 2:** Top 20 search queries in Japan.

Search queries (English translation)	Average search volume per month	Four-year total search volume (per 100 people)	Total hit websites	Association website rank
zakotsushinkeitu (sciatica)	135,000	6,250,000 (4.94)	5,870,000	13
youtuu (lower back pain)	90,500	4,737,000 (3.74)	83,300,000	2
rokkanshinkeitsu (intercostal neuralgia)	74,000	3,724,500 (2.94)	577,000	>100
kensyouen (tendosynovitis)	74,000	3,307,000 (2.61)	8,660,000	3
komuragaeri (leg cramps)	49,500	2,217,300 (1.75)	995,000	>100
senaka no itami (back pain)	40,500	2,120,800 (1.68)	26,600,000	91
koshi ga itai (my lower back aches)	40,500	2,103,600 (1.66)	35,900,000	88
hiza no itami (knee pain)	40,500	2,090,900 (1.65)	25,000,000	7
ashi no ura itai (sole pain)	40,500	2,081,400 (1.64)	26,800,000	95
senaka ga itai (my back aches)	33,100	1,721,300 (1.36)	28,000,000	>100
hiza ga itai (my knee aches)	33,100	1,670,600 (1.32)	19,000,000	23
senaka itai (back, pain)	33,100	1,635,300 (1.29)	30,100,000	>100
kubi ga itai (my neck aches)	33,100	1,580,000 (1.25)	38,800,000	7
gojukata (painful shoulder at age 50s)	33,100	1,476,200 (1.17)	8,650,000	2
henkeiseihizakansetsusho (osteoarthritis of the knee)	33,100	1,413,600 (1.12)	4,860,000	2
kokansetsu itami (hip, pain)	27,100	1,310,700 (1.04)	7,360,000	5
shinkeitsu (neuralgia)	27,100	1,224,600 (0.97)	7,660,000	17
fukurahagi itai (calf, pain)	22,200	1,163,900 (0.92)	1,900,000	8
hiza itami (knee, pain)	22,200	1,139,400 (0.90)	26,600,000	4
shijukata (painful shoulder at age 40s)	22,200	1,115,300 (0.88)	253,000,000	>100

## Discussion

This study is the first to examine how frequently the patient information pages of orthopedic associations appear when search terms related to musculoskeletal pain are entered. Based on the results of this study, we believe that there is a need for orthopedic associations to do more to provide medical information on the internet by optimizing their position in the search results for frequently searched symptom and disease-related terms.

The click-through rate for the first position in Google was 21.12%, followed by 10.65% and 7.57%. If the search result ranked 11th, the click-through rate became only 1.46% [15]. In a German study, when 289 patients searched for medical information on the internet, only 5 clicked a link on the second or subsequent search result pages [16].

In the early 2000s, over 60% of people in the United States and Australia reported that they had used the internet to search for medical information and were satisfied with the resulting health information and services [17-19]. More recently, 96% of people in Australia reported searching for medical information online; of those, 63% searched using smartphones [20]. Over time, it has become increasingly common for individuals to use online search engines when they need medical or health information.

According to many reports, inaccurate websites often appear among the top 10 results and provide erroneous information [21,22]. In the United Kingdom, Wikipedia sometimes appears more often in the search results than the National Health Service’s website [23]. If members of the general public encounter websites containing incorrect information, it is difficult for them to assess its accuracy. In a US study, high school students with a science-focused education had difficulty distinguishing between reliable and unreliable medical sites, even though most of the information produced in the searches was inaccurate [24]. To solve health problems, government organizations need to work together to provide more accurate medical information on the web [9].

The algorithm used for Google searches is confidential; thus, many websites try to increase the quantity and quality of their traffic using organic search engine results. This practice is termed search engine optimization (SEO). At present, if items do not appear among search engine results, they are practically nonexistent.

In this study, we surveyed the search rankings of the patient information pages of two orthopedic associations and found that these pages are not likely to be viewed by patients seeking medical information with frequently searched queries. There was no significant difference in the number of searches per 100 people between the United States and Japan in the top 20 pages retrieved for musculoskeletal-related pain. Symptoms and illnesses are searched differently between the United States and Japan. The common query “leg cramps” was searched much more often in Japan per 100 people and was searched more often in the summer in both the United States and Japan (Figure 2). The predominance in summer suggests a link to climate. Indeed, in the United Kingdom and Australia, the volume of searches related to leg cramps increased in summer [25]. Another study found that searches related to ankle swelling also increased in midsummer [26]. Heartburn-related searches increased during winter, as did heart failure hospitalizations [10,27]. These studies indicate that an increase in the search volume for a particular condition reflects an actual increase in the number of patients affected with that condition. Thus, when the search volume increases, it may be effective for medical associations to provide medical information to patients who need accurate information using social networking sites such as Facebook and Twitter. The act of conducting medical interventions via social media (ie, social media intervention) is reported to be an effective tool for enhancing public health [28]. In the future, it will be important to increase interventions in addition to disseminating medical information via social networking sites. The AAOS has a Twitter account, whereas the JOA currently does not (as of June 2020).

On December 6, 2017, Google made an official announcement about improving the quality of search results for medical and health matters [29]. After this statement was made, information from health professionals, specialists, and medical institutions should have become more readily available, and information sources such as blogs and websites dealing with folk medicine should have been excluded from searches. However, the websites of the two associations we focused on rarely appeared among the top 10 results, indicating that no dramatic change has in fact taken place. In that announcement, Google stated that health experts should make their websites appropriate for general users by avoiding the use of technical terms. Fortunately, personal blogs and unofficial websites that may have posted incorrect medical information tended not to appear in the search results owing to Google’s corporate efforts.

The findings of our study suggest that better SEO is required to boost the ranking of medical associations’ websites. If their websites were to achieve the highest ranking, ordinary patients could more readily obtain appropriate medical information. Of course, it is also necessary to include accurate medical information on the associations’ patient information pages. An SEO strategy comprises two categories: on-page and off-page SEO. Details about this may be found in the Google Search Engine Optimization Starter Guide [30] and the Google Webmaster Guidelines [31]. Unfortunately, development of an SEO takes time. Daily updates and accurate articles need to be posted online to raise the rankings of the medical associations. In the future, a dual approach may be necessary, involving passive information transmission through companies such as Google, and active information transmission using social networking services such as Twitter and Facebook.

## Abbreviations

AAOS: American Academy of Orthopedic Surgeons
JOA: Japanese Orthopedic Association
SEO: search engine optimization
